# MAPLE Assembled Acetylcholinesterase–Polyethylenimine Hybrid and Multilayered Interfaces for Toxic Gases Detection

**DOI:** 10.3390/s18124265

**Published:** 2018-12-04

**Authors:** Valentina Dinca, Cristian Viespe, Simona Brajnicov, Izabela Constantinoiu, Antoniu Moldovan, Anca Bonciu, Constantin Nicolae Toader, Raluca Elena Ginghina, Nicoleta Grigoriu, Maria Dinescu, Nicu Doinel Scarisoreanu

**Affiliations:** 1 National Institute for Lasers, Plasma and Radiation Physics, Magurele, Bucharest 077125, Romania; valentina.dinca@inflpr.ro (V.D.); brajnicov.simona@inflpr.ro (S.B.); izabela.constantinoiu@inflpr.ro (I.C.); antoniu.moldovan@inflpr.ro (A.M.); anca.bonciu@inflpr.ro (A.B.); maria.dinescu@inflpr.ro (M.D.); nicu.scarisoreanu@inflpr.ro (N.D.S.); 2 Scientific Research Center for CBRN Defense and Ecology, Bucharest 041309, Romania; cntoader@yahoo.com (C.N.T.); ginghinaraluca@gmail.com (R.E.G.); nico.grigoriu@gmail.com (N.G.)

**Keywords:** MAPLE, surface acoustic wave sensor, AchE, DMMP detection, DIMP detection

## Abstract

Developing a controlled method for obtaining hybrid enzymatic-based interfaces for sensing application require the use of a multiuse, reusable sensor. By controlling the interface characteristics in terms of the surface chemistry, thickness, and roughness, a tailored response toward various toxic compounds can be obtained, regarding both materials used as active surfaces and fabrication methods. Herein, we report a preliminary study on using a laser-based method (i.e., matrix-assisted pulsed laser evaporation, or MAPLE) for obtaining active polymeric–enzymatic interfaces as hybrid or layered coatings for detecting toxic vapors. The MAPLE fabrication consisted of the simultaneous alternating evaporation of layers of polyethylenimine (PEI) and acetylcholinesterase (AchE) in order to obtain active surfaces as both hybrid PEI-AchE and a PEI/AchE layered coating, respectively. The deposition processes of the polymer and enzyme were carried out using a double-target system and a Nd:YAG pulsed laser, operating at 0.45 J/cm^2^ fluences with a wavelength of 266 nm and a repetition rate of 10 Hz. Fourier transform infrared spectroscopy revealed no significant changes in the functional groups of both hybrid and layered coatings compared with the initial material. The thickness and roughness, as well as the morphologies of the coatings revealed by atomic force microscopy and scanning electron microscopy showed coatings thicker than two μm that had smooth surfaces and average roughness values below six nm. The sensors were tested with simulants for nerve gases and pesticides containing phosphonate ester groups, namely dimethyl methylphosphonate (DMMP) and diisopropyl methylphosphonate (DIMP), and a different sensitivity was shown to the selected chemical agents for each of the sensors. The best sensitivities for DMMP and DIMP obtained by using a PEI-AchE coated sensor are 65 kHz and 200 kHz, respectively, whereas the best sensitivity when using multilayered interfaces is 30 kHz and 10 KHz for DIMP and DMMP, respectively.

## 1. Introduction

Nowadays, the fast, sensitive detection of specific harmful chemical agents presents interest as a research topic, which is caused by security concerns and safety hazards. Therefore, detecting quantities lower than those that can affect negatively life and health, as well as discriminating between chemical toxic compounds is of interest for wide area of applications related to health and security.

Among the most used sensors, surface acoustic wave (SAW) sensors are used at a larger scale for research related to the detection of volatile toxic compounds due to specific characteristics: high sensitivity, small size, low cost, very good response time, and ability to work in wireless mode.

The SAW sensor sensitivity and selectivity are related both to sensor characteristics and active area interface properties. This implies the use of specific compounds and methods that are compatible with them, which allow tailoring their characteristics [1,2,3,4,5,6,7].

The organophosphorus compounds have a significant negative impact as related to terrorism. Such compounds are also environmental and food chain pollutants (i.e., DMMP, or dimethyl methylphosphonate), as they are used as common additives for anti-foaming agents, plasticizers, stabilizers, textile conditioners, and antistatic agents [8]. Moreover, DMMP is used due to its nontoxicity and organophosphorus compound elemental composition for mimicking nerve agents, being considered an appropriate simulant for both insecticides and G-series nerve agents. Besides DMMP, diisopropyl methylphosphonate (DIMP) can be used as well as simulant for G-series nerve agents.

Therefore, one major requirement for obtaining highly sensitive active elements for sensors is the synergy between specific functional materials with advanced fabrication technology. Polyethylenimine (PEI) and acetylcholinesterase (AchE) were chosen for obtaining the hybrid and multilayered coatings due to their peculiar characteristics suitable for sensing DMMP and DIMP. Specifically, among the polyamines, polyethyleneimine (PEI) represents one of the most interesting candidates for binding specific volatile compounds due to its high amine density and accessible primary amine sites on the chain ends (for example, for CO_2_ capture ability, etc.) [9]. In the literature, AchE immobilized through physical adsorption is mainly used for biosensors, especially for testing/screening therapeutic drugs for Parkinson’s and Alzheimer’s diseases, as well as in clinical diagnosis [10,11,12,13]. For this direction, films of silica sol-gel incorporating gold nanoparticles (AuNPs-Si-SG) coated with AchE, or platinum-coated with AchE, were used to test various drugs [14].

The use of active coatings with tunable characteristics in sensing fields is directly correlated with the surface chemical and topographical properties. Therefore, the method of preparation, and the type of analytes implied must to be correlated to the specific application. 

There are chemical methods and physical methods that can be used to modify/coat a surface; this involves processes from adding suitable functional groups on the surface (i.e., chemical vapor deposition (CVD)) to adding physical material onto a surface (i.e., spin coating, dip coating, vapor deposition, sputtering, arc vapor deposition, and ion plating). If in the case of CVD, the thickness of the film can be controlled even to an atomic level, but the precursors are highly toxic, corrosive, or explosive, causing the destruction of the biocompounds or adverse toxic effects. In the case of spin coating and dip coating, it is difficult to control the thickness of the film, and no hybrid materials that imply organic solvents and proteins/enzymes can be obtained [15].

In the last years, matrix-assisted pulsed laser evaporation (MAPLE) was successfully used for depositing sensitive materials such as polymers and proteins, but it was also shown to be an appropriate approach for embedding in a controlled manner not only ceramic materials or graphene, but also active proteins such as lactoferrin, or, for a narrow window of parameters, functional Micrococcus bacteria for biosensing applications [16,17,18,19,20,21,22]. 

MAPLE provides a suitable process for transferring various small or large molecular weight species as coatings from the condensed phase into the vapor phase. The process starts with the laser energy being mostly absorbed by the solvent molecules, therefore preventing the target molecules from being damaged by the high-energy laser beam. The solvent vaporization mechanism includes the photo thermal process that converts the absorbed energy of the photons from the frozen solvent molecules to thermal energy [15]. Therefore, when “target molecules absorb enough energy through collisions with solvent molecules under the evaporation process, the target molecules are transferred to the vapor phase”. It is important to underline that the MAPLE target solution is depleted layer by layer; the concentration does not change within the experiment time [15]. Depending on the experimental conditions, and due to the low solvent adhesion coefficient, most of the solvent molecules are pumped away [15,16,17,18,19,20,21,22]. When it is necessary to obtain coatings from not miscible elements, targets can also be designed as a multi-compartment system, therefore allowing a unique method to “mix” proteins with polymers that use organic solvents [18]. 

Within this context, in this work, the matrix-assisted pulsed laser evaporation (MAPLE) technique was used to yield hybrid PEI-AchE, which can form respectively layered PEI/AchE thin films for efficient DMMP and DIMP capture. In previous work by Viespe et al. [23], it was observed that if the PEI is deposited by air-brush technique, the film morphology is affected, with direct consequences on the signal-to-noise ratio (SNR). 

In this work, MAPLE was used to evaporate simultaneously or alternatively layers of PEI and AchE. Micron-thick sensitive layers formed active surfaces: hybrid PEI-AchE and PEI/AchE layered coatings, repsectively. These were tested with DIMP and DMMP. Our approach presents not only the advantage of controlling the morphology of the polymer and enzyme films deposited on quartz substrates, but, more importantly, when using the MAPLE technique, the solvent used for the PEI polymer will not contact the AchE enzyme; therefore, it will not affect its functionality.

## 2. Materials and Methods

### 2.1. Target Solutions Preparation

The chemicals were obtained from Sigma-Aldrich (Saint Louis, MO, USA). Deionized water and methanol were used as solvents for AchE and PEI, respectively. Solutions of 2% weight PEI (408719 Aldrich, average Mw ~800 by LS, average Mn ~600 by GPC) in methanol, and 0.1% weight AchE (C3389 Type VI-S, lyophilized powder, 200–1000 units/mg protein) in double-distilled water were obtained. The PEI solutions were subsequently sonicated for several minutes (30 min) (Sharpertek Digital Ultrasonic cleaner XP PRO).

### 2.2. Matrix-Assisted Pulsed Laser Evaporation System

**Figure 1:**
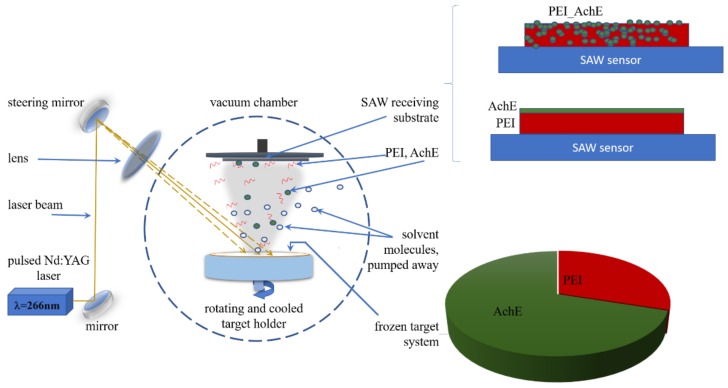
Matrix-assisted pulsed laser evaporation (MAPLE) setup and target system used for obtaining hybrid polyethylenimine (PEI)-acetylcholinesterase (AchE) coatings and layered PEI/AchE thin films.

A “Surelite II” pulsed Nd:YAG laser system (Continuum Company) (five to seven ns pulse duration) at 266 nm and a 10-Hz repetition rate was used to irradiate the frozen targets. In order to avoid the influence of methanol on AChE, we used a double-target system, consisting of 75% area occupied by AchE and 30% occupied by PEI (Figure 1). In order to form the target, the solutions were a sonicated for five minutes and rapidly frozen in a liquid nitrogen-cooled copper container. Firstly, the PEI solution was confined within 30% of the target by using a removable Teflon separator; secondly, the AchE solution was frozen, and the teflon separator was removed. The container was mounted on a cryogenic holder inside the deposition chamber (Neocera spherical vacuum chamber with 12” diameter). The target was maintained frozen by a circulating liquid nitrogen system; the temperature was checked by the two thermocouples placed directly onto the target holder. The laser fluence was 0.45 J/cm^2^ and the number of pulses was kept at 54 k pulses (for AchE) and 36 k pulses (for obtaining a 2.4-um thick PEI and a 200-nm thick PEI-AchE enzyme coatings). Also, in order to avoid damage by local overheating and drilling following multiple pulses of laser irradiation, the target was rotated with 20 rpm using a motion feed-driven motor. Due to the absorbed laser energy in the first frozen layer of the target, which consisted mostly of frozen solvent, vaporization takes place in order to entrain the enzyme and polymer particles toward the substrate, while volatile solvent molecules were removed from the deposition chamber by the vacuum pumps. The substrates are placed parallel to the target and situated at a distance of 3.5 cm. The background pressure in the chamber was maintained at 1–2 × 10^−3^ Pa.

The hybrid PEI-AchE coatings were obtained by simultaneously scanning the laser beam onto the dual target; within the same experiment, respectively layered PEI/AchE thin films were obtained by firstly scanning the laser beam onto the PEI target, and secondly onto the AchE target surface (Figure 1).

### 2.3. Substrate Preparation

Two types of substrates were used: double-polished Si (100) transparent in the infrared (Neyco), and SAW sensors.

The Si substrates that were used for Fourier transform infrared (FTIR) measurements, as well as for atomic force microscopy (AFM) and SEM were cleaned by sonication with alcohol and water and blow-dried under N_2_ gas before use. All of the substrates were placed at a distance of 3.5 cm from the frozen target and kept at ambient temperature during the deposition.

The SAW sensors were fabricated on ST-cut quartz with propagation in the X-direction. The interdigital transducers consisted of 200 nm of gold on 10 nm of chromium as an adhesive layer. A double-double finger design was used with a periodicity of 11 µm. The SAW sensors’ operation frequency was ~69 MHz. For the measurement circuit, a DHPVA-100 FEMTO (10–60 dB, 100 MHz) amplifier was used, and the frequency shift of the system was read using a CNT-91 Pendulum counter analyzer [23,24]. 

### 2.4. Chemical and Morphological Characterization of the Deposited Thin Films

Fourier transform infrared spectroscopy (FTIR) was used to evaluate the characteristic vibrations of the functional groups of the substrate-deposited thin films. The infrared spectrum of the native molecule deposited by drop cast on the Si substrates was used as the control. The FTIR measurements were carried out using a Jasco FT/IR-6300 type spectrometer in the 400–4000 cm^−1^ range, with a resolution of 4 cm^−1^. The spectra were measured by transmission through a coated Si wafer, and then the absorption was calculated by the accumulation of 1024 scans. For maintaining a steady atmosphere in the measurement chamber, silica gel and regularly purging the spectrometer with argon gas were used. The employed substrate that was used as the background as well was a thin silicon wafer (transparent to infrared).

Morphology characterizations were performed by optical microscopy, atomic force microscopy (AFM), and scanning electron microscopy (SEM). For optical microscopy, the images were acquired using an Axiovert 200 Microscope coupled to a Carl Zeiss AxioCamMRm camera. AFM (XE 100 AFM, Park systems) measurements were performed in non-contact mode, and allowed for surface roughness analyses. 

SEM investigations were carried out on a field emission scanning electron microscope (JSM-531 Inspect S Electron Scanning Microscope, FEI Company (Hillsboro, OR, USA)).

### 2.5. DMMP and DIMP Measurements

The different concentrations of DMMP and DIMP were detected using a testing system developed by Viespe et al. [25]. The DMMP and DIMP liquid was injected into a gas mixture and mixed with air. The amount of DMMP and DIMP, as shown in Table 1, were six ppm and five ppm respectively, after the total evaporation of the analyte that was circulated in the system by a diaphragm pump (Pfeiffer model MVP 035-2). The mixture temperature and the flow rate were maintained constant during the experiments. Repeating 10 measurements of the frequency deviation for each of the sensor films yielded errors below as ± 4%.

## 3. Results and Discussion

There are various surface and interface characteristics of an active sensor element (i.e., thickness, uniformity, roughness, chemistry) that can influence and dictate the response of a sensor to a specific analyte. By the ability to control and tailor both the physical and chemical characteristics of the interfaces, an enhanced response and use of these active coatings can be obtained. In this work, we used the MAPLE technique for depositing hybrid and layered active surfaces based on PEI and AchE for detecting DMMP and DIMP, respectively.

### 3.1. Morphological Characterization

**Figure 2:**
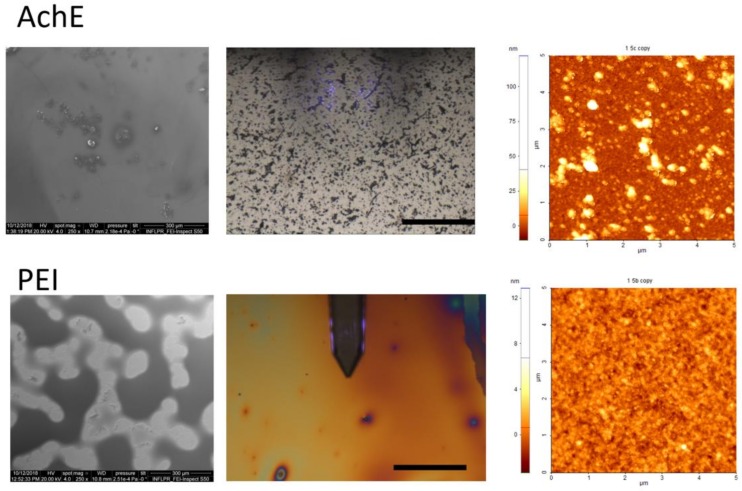
SEM, optical microscopy, and atomic force microscopy (AFM) images of AchE and PEI drop-casted materials.

**Figure 3:**
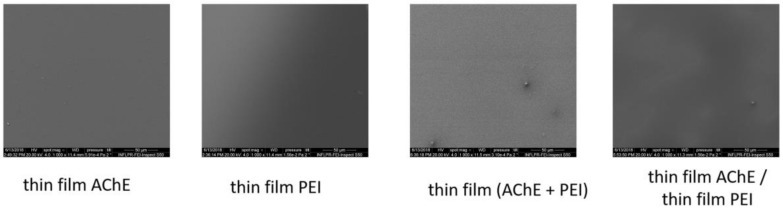
Top-view SEM of the MAPLE samples obtained on Si. Scale bar: 50 μm.

**Figure 4:**
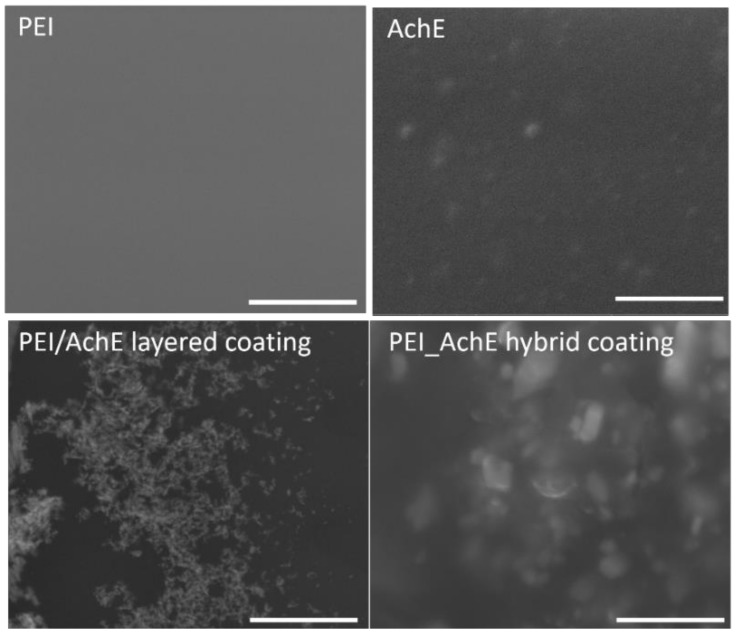
SEM images (with 20,000 magnification) of the samples obtained by MAPLE. (Scale bars: four μm).

**Figure 5:**
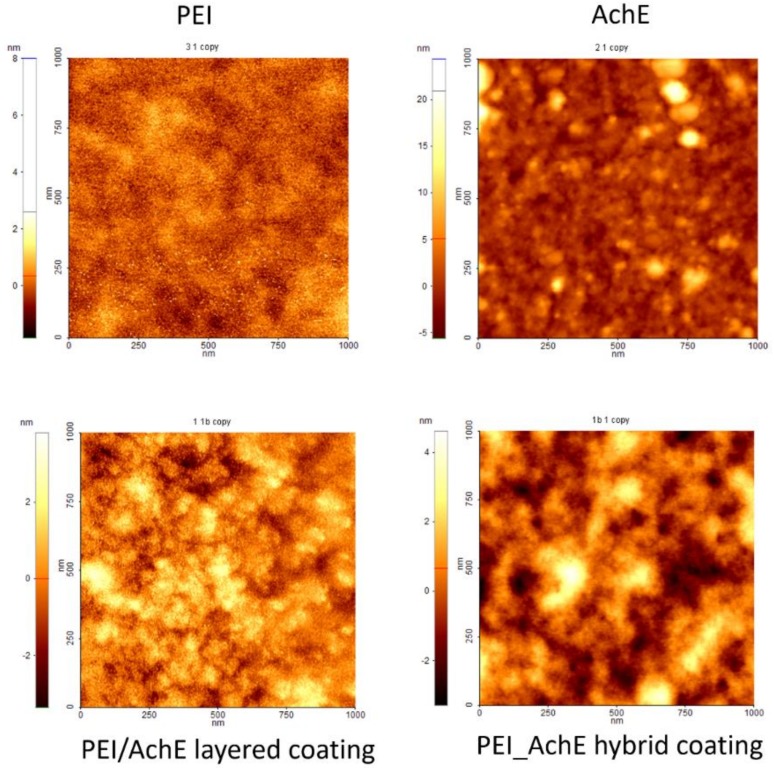
The AFM analysis of the MAPLE-coated samples (1 μm × 1 μm).

Scanning electron microscopy and atomic force microscopy were used first to analyze the morphology and roughness of the deposited and drop-casted components of the layered and hybrid coatings surfaces with a final thickness of 2.4 μm in different areas of the samples. Examples of the SEM and AFM images of the samples obtained by both drop cast and MAPLE are presented in Figure 2 (for the drop-casted material), and in Figure 3, Figure 4 and Figure 5 for the samples obtained by MAPLE. 

It was observed that the control samples, which were obtained by drop casting, as shown in Figure 2, are characterized by relatively smooth surfaces, but AchE presented an irregular accumulation of material as nanograins onto the surface. The optical microscopy images show a larger accumulation of these grain-like structures over the entire area of the samples. The irregular shapes are present in the case of PEI too; the material is spread non-uniformly. 

In contrast with the control samples, the main characteristics for the MAPLE-obtained samples, as confirmed by both SEM and AFM analysis, are uniformity and low roughness surface, as can be seen for the PEI, AchE, and PEI/AchE thin films (Figure 3, Figure 4 and Figure 5). 

However, it was observed as well that in the case of layered coatings, the protein can accumulate, leaving part of the PEI exposed. However, in the case of hybrid coatings, the proteins deposit seems to be embedded within the polymeric layer. 

For a better visualization of the surface topography and understanding of the material organization on the surfaces, AFM measurements were performed, showing slightly porous surfaces, with pores varying within tens of nanometers in the case of hybrid PEI_AchE coatings (Figure 5). The non-contact mode AFM images showed smooth structures of both the single elements and hybrid surfaces; roughness levels below six nm were observed (i.e., 2.2 nm for PEI, 4 nm for AchE, 2.8 nm PEI/AchE, and 5.9 nm for PEI_ACHE).

### 3.2. Chemical Characterization

**Figure 6:**
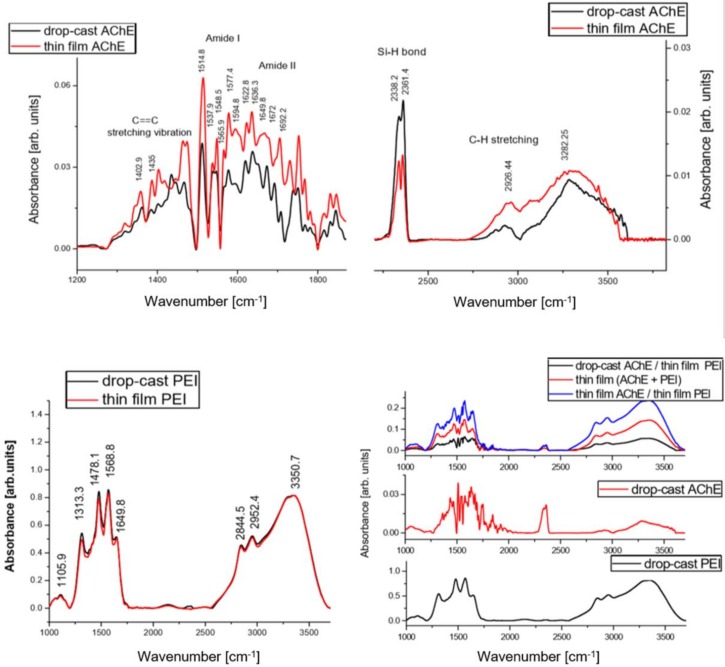
FTIR spectra of PEI polymer films, AchE enzyme, and PEI-AchE hybrid coatings, deposited by MAPLE and the drop-casted solution as a reference of the peaks positions and widths.

The differences induced by deposition methods (MAPLE and drop cast) in the functional groups among the PEI, AchE, PEI-AchE, and PEI/AchE samples were determined by FTIR measurements. The similarity between the absorbance bands for drop cast and the thin films obtained by MAPLE are depicted in Figure 6 and Table 1. The significant absorption regions of the deposited material and control are shown, allowing the optimum visualization of the peaks and the changes induced by the MAPLE process.

The composition was preserved after dissolving the PEI in methanol (drop cast), as well as for MAPLE transfer (Figure 6). The unmodified functional groups of PEI were indicated by the signals at 3500 cm^−1^ corresponding to NH asymmetric stretching, and at 3391 cm^−1^ and 3365 cm^−1^, corresponding to NH symmetric stretching. The CH symmetric and asymmetric stretching were confirmed by the signals from 2881 cm^−1^ and 2915 cm^−1^, respectively. The N–H deformation was confirmed at 1647 cm^−1^, while the peaks observed at 1496 cm^−1^ and 1043 cm^−1^ corresponded to C–H deformation and C–N stretching, respectively [25].

Moreover, the functional groups of AchE samples obtained by MAPLE were indicated by the signal at 3282.25 cm^−1^, corresponding to AchE’s free hydroxyl stretching mode [26,27,28,29]. Due to adsorbed hydrocarbons on the surface, the C–H stretching vibrations are confirmed by the signal at 2926 cm^−1^. Although the samples were maintained through using silica gel and purging the spectrometer with argon gas, the CO_2_ interferences from ambient conditions are noticed as a double peak located at 2340 cm^−1^ and 2360 cm^−1^ (asymmetric stretching). The signals at 1641 cm^−1^ were associated with the NH bending and scissoring mode, while the signal in the range 1451–1410 cm^−1^ was assigned to the C==C stretching vibration arising from the deposited enzyme on the surface [26,27,28,30,31]. Furthermore, the spectrum of MAPLE-deposited AchE-PEI hybrid coatings showed absorptions similarities with that of the drop-casted samples for both components. However, as previously reported, the presence of enzyme immobilized onto a surface was confirmed by the observation of broad and intense bands for amide I at ~1655 cm^−1^ (νC=O stretching vibrations) and amide II at ~1530 cm^−1^ (combination of δN−H bending and νC−N stretching modes) [28], while in our case, despite the similarity between the drop-cast spectra and the MAPLE spectra, the peaks corresponding to amide I were observed at 1641 cm^−1^, while the Amide II band was observed at 1514 cm^−1^. If in the works reported previously, the bands follow the general trend of displaying broad unstructured bands without any overlaid fine structures, in our case, several components bands are observed at 1636 cm^−1^, 1649 cm^−1^, 1672 cm^−1^, and 1690 cm^−1^. As reported by Gorne-Tschelnokow et al., by analyzing the deconvoluted spectra of the enzyme, components were observed as well at 1631 cm^−1^, 1648 cm^−1^, and 1656 cm^−1^; meanwhile, weaker bands appeared near 1622 cm^−1^, 1640 cm^−1^, and 1672 cm^−1^ [26]. Other minor peaks (between 1700–1850 cm^−1^) are related to the carbonyl stretching absorption. Also, as previously observed in the work of Khaldi et al. [28], the peaks observed in the region of 1400−1200 cm^−1^ are assigned to amide III (νC−N stretch and νN−H bend near 1300 cm^−1^). 

These observations demonstrate a non-destructive laser transfer of the PEI and AchE thin film, without methanol solvent molecules on the substrate or interfering with AchE after MAPLE deposition, allowing the deposition of two different configurations for the active element of the sensor, as both layered and mixture/hybrid coating.

### 3.3. DMMP and DIMP Measurements

A comparison between the frequency shifts values for the coated sensors with either PEI or hybrid and layered coatings, together with the effects of the presence of DMMP and DIMP, is shown in Table 2. An increase of the value of frequency shift—meaning a better response for DIMP—was observed for all of the samples, while hybrid PEI-AchE coatings gave a better response as compared with both PEI and PEI/AchE layered coatings. The frequency shift that was obtained for a DMMP concentration by us was better than the results obtained with SAW sensors using ZnO [29] and polysiloxane [32] for DMMP detection. The response time was between 9–15 s and 90–100 s in the case of DIMP and DMMP, respectively. This response could be explained by the vapor pressure of DMMP and DIMP. DMMP has a higher vapor pressure at 25 °C (0.962 mmHg) than DIMP (0.28 mmHg), which makes it evaporate over a longer time in site.

By comparing the results for the three types of sensors, one can notice a difference in frequency change depending on the type of film deposited. Thus, for the PEI film, where interaction took place through weak hydrogen bonds [33], the lowest values were obtained. For the layered film, where the enzyme interacts directly with the analyte, the results are intermediate. In this case, the signal is received by an effect, π, (between the electrons p of the nitrogen and the electrons π of the phosphate group), providing stronger bonds than the hydrogen bonds formed by PEI [34,35,36].

The better response given by the hybrid coating can be explained by the synergistic effect of PEI and AchE, with both the polymer and the enzyme acting in the presence of DMMP and DIMP, through weak hydrogen bonds and the p–π effect. Also, the AchE is well-known for providing a binding site through the exposure of both an esteratic subsite (Ser-His-Glu) and peripheral binding anionic subsite that has the ability to bind to many different types of ligands [37]. Moreover, as shown in Figure 4, the hybrid surfaces are also characterized by the presence of nm-sized pores that could lead to increasing the active surface area of the sensor. Nevertheless, the differences between the results for DIMP and DMMP can be explained by the electron repellent inductive effect of the methoxy groups and the methyl radicals, which form a larger electronic cloud on the DIMP phosphorus than the DMMP [38]. 

These non-covalent interactions of this system with DMMP could also represent the basis for the realization of reversible detectors of nerve agent simulants in solution and also on solid supports. Nevertheless, as sensitivity and selectivity are crucial factors within sensor applicability, the perspective envisages discriminating volatile nerve agents and/or other toxic organophosphates compounds from non-toxic substances in a complex gaseous environment by using sensor array based on SAW resonators and active elements based on AchE, but in combination with different types of chemoselective polymers that could give the system both selectivity and sensitivity toward various chemical agents.

## 4. Conclusions

This study demonstrated the feasibility of obtaining hybrid polymer–enzyme active interfaces for PEI-AchE sensors by using MAPLE and a modular target system. The two different configurations of PEI-AchE depositions were characterized by SEM and AFM, and revealed smooth surfaces, except for the presence of nm-sized pores in the case of the hybrid surfaces. Moreover, no significant modifications of FTIR spectra were observed either after MAPLE deposition or AchE inclusion within the PEI polymer. On the other hand, the inclusion of AchE within the PEI coating played a major role in increasing the measurement sensitivity for both DIMP and DMMP detection. This demonstrates that the enzymes and polymer deposited by MAPLE can provide good premises for the development of a sensor with suitable stability, good reproducibility, and high sensitivity toward DMMP and DIMP. 

Such a laser-based coating deposition approach demonstrates the great potential of PEI-AchE-based enzymatic interfaces in a sensing system, with potential perspectives to be used for biomedical and clinical diagnostic applications.

## Figures and Tables

**Table 1:** Fourier transform infrared (FTIR) measurements.

Position (cm^−1^)	Vibrations
PEI	
3500	NH asymmetric stretching
3391	NH symmetric stretching
3365	NH stretching II
2915	CH asymmetric stretching
2881	CH symmetric stretching
1647	N–H deformation
1496	C–H deformation
1043	C–N stretching
AchE	
3282.25	OH stretching
2962	C–H stretching
2340; 2360	Asymmetric stretching, CO_2_ gas phase influence
1700–1600	Amide I
1600–1500	Amide II
1451–1410 1200−1405 1300	C==C stretching vibration νC−N stretch νN−H bend

**Table 2:** Frequency shift of sensors at six-ppm dimethyl methylphosphonate (DMMP) concentration and five-ppm diisopropyl methylphosphonate (DIMP) concentration.

Sensor Type	DIMP	DMMP
	Frequency Shift [kHz]	Frequency Shift [kHz]
Multilayer PEI/AchE	30	10
PEI with AchE	200	65
PEI	11	4
